# Silica Nanoparticles from Coir Pith Synthesized by Acidic Sol-Gel Method Improve Germination Economics

**DOI:** 10.3390/polym14020266

**Published:** 2022-01-10

**Authors:** Josef Maroušek, Anna Maroušková, Rajiv Periakaruppan, G. M. Gokul, Ananthan Anbukumaran, Andrea Bohatá, Pavel Kříž, Jan Bárta, Pavel Černý, Pavel Olšan

**Affiliations:** 1 Institute of Technology and Business in Czech Budejovice, Faculty of Technology, 370 01 České Budějovice, Czech Republic; anita.tbs@gmail.com; 2 Faculty of Management and Economics, Tomas Bata University in Zlin, 76001 Zlín, Czech Republic; 3 Faculty of Agriculture, University of South Bohemia in Czech Budejovice, 37005 České Budějovice, Czech Republic; abohata@centrum.cz (A.B.); kriz@pf.jcu.cz (P.K.); barta@zf.jcu.cz (J.B.); pcerny@pf.jcu.cz (P.Č.); olsan@zf.jcu.cz (P.O.); 4 Department of Biotechnology, Karpagam Academy of Higher Education, Coimbatore 641021, India; rajivsmart15@gmail.com (R.P.); gokulgmgk@gmail.com (G.M.G.); 5 Department of Microbiology, Urumu Dhanalaksmi College, Tiruchirapalli 620019, India; anbhu34526@gmail.com; 6 Department of Applied Physics and Technology, Faculty of Education, University of South Bohemia, 37115 České Budějovice, Czech Republic

**Keywords:** silica nanoparticles, coir pith, sustainability, phytochemical analysis, bioeconomy

## Abstract

Lignin is a natural biopolymer. A vibrant and rapid process in the synthesis of silica nanoparticles by consuming the lignin as a soft template was carefully studied. The extracted biopolymer from coir pith was employed as capping and stabilizing agents to fabricate the silica nanoparticles (_n_Si). The synthesized silica nanoparticles (_n_Si) were characterized by ultraviolet–visible (UV–Vis) spectrophotometry, X-ray diffraction analysis (XRD), Scanning Electron Microscope (SEM), Energy-Dispersive X-ray Analysis (EDAX), Dynamic Light Scattering (DLS) and Fourier-Transform Infrared Spectroscopy (FTIR). All the results obtained jointly and independently verified the formation of silica nanoparticles. In addition, EDAX analysis confirmed the high purity of the _n_Si composed only of Si and O, with no other impurities. XRD spectroscopy showed the characteristic diffraction peaks for _n_Si and confirmed the formation of an amorphous nature. The average size of _n_Si obtained is 18 nm. The surface charge and stability of _n_Si were analyzed by using the dynamic light scattering (DLS) and thus revealed that the _n_Si samples have a negative charge (−20.3 mV). In addition, the seed germination and the shoot and root formation on *Vigna unguiculata* were investigated by using the _n_Si. The results revealed that the application of _n_Si enhanced the germination in *V. unguiculata*. However, further research studies must be performed in order to determine the toxic effect of biogenic _n_Si before mass production and use of agricultural applications.

## 1. Introduction

There is a broad consensus that the nanoparticle is a material with at least one dimension less than 100 nm. Nanoparticles can be distinguished into nanopowders, nanoclusters, nanocrystals and many other groups which can be further subdivided [1]. At the end of the 20th century, nanotechnology was perceived as the next game-changer [2]. Based on the laboratory experiments, as more and more nanomaterials of different compositions, sizes and shapes became available [3], dramatic changes were predicted to improve human lives [4]. Nanomaterials showed varied optical, catalytic, magnetic and other chemical–physical characteristics, including distinct biological properties, such as antimicrobial and anti-inflammatory activities [5]. Most of these excellent properties have been repeatedly and independently confirmed in the chemical industry [6], metal production [7], agriculture [8] and energetics [9] (Mardoyan and Braun 2015), to name a few. However, as fast as the nano industry grew initially, it hit its upper limit about a decade ago, and a price ceiling has been slowing down its further development since then [10]. A plethora of methods have been developed to synthesize various nanomaterials of different characteristics. The two most important production directions are A/electrochemical and chemical reduction [11] and B/photochemical and physical vapor condensation [12]. Carbon nanotubes, quantum dots, nanorods, nano capsules, nano emulsions, fullerenes, metallic nanoparticles, ceramic nanoparticles and polymer nanoparticles hold the largest market share [13,14], whereas usual wholesale prices range from 4 to 18 €g^−1^ [15]. Although these conventional production processes make it possible to achieve nanoparticles with perfect shapes and a purity higher than 99.995%, it is the high production costs (about 90% of the market price) that block further industry development [16,17]. To make matters worse, all of these various combinations of chemical and physical methods are energy demanding and require hazardous reagents (mostly stabilizing and reducing agents) during almost all production phases [18], not to mention various biological risks to the environment [19]. Hence, there is a wide demand for the definition of less demanding production technologies that would improve the competitiveness of the entire nanotechnology industry [20].

Si and SiO_2_ nanomaterials have drawn more attention by various entrepreneurs due to their widespread application in the advance of new technologies in various areas [21,22]. They have a wide range of applications in industries such as agriculture, pharmacy, pigments, catalysis, electronics and cosmetics [23,24]. There are numerous types of _n_Si, including non-porous, mesoporous, hollow mesoporous and core–shell, all of which can be modified on the surface [25,26]. Mesoporous _n_Si have few flexible and desirable properties, such as biocompatibility, tenable pore size and volume for delivery of targeted drugs [27]. Using Tetra ethyl ortho silicate [Si(OC_2_H_5_)_4_,TEOS] as a precursor is the most straightforward and cost-effective method for producing spherical, monodispersed and nanosized _n_Si [28]. In plants, silica is important for inducing resistance against the biotic and abiotic stresses [29]. The recent advances in nanotechnology and its use in agriculture fields are astonishingly increasing to improve crop production [30].

There are various methods, namely Sol-Gel, reverse microemulsion and flame-synthesis methods employed in extracting silica from waste materials. The Sol-Gel method is the most common approach for research purposes. The original method of Stöber et al. [31] has largely altered and modified the synthesis of silica via hydrolysis–condensation reaction. The polymeric networks of gels were formed from silicon alkoxide/halide gels, and polymeric gel is otherwise known as as xerogel [32]. Many silica-based nanomaterials and derivatives are produced by using the Sol-Gel method. The acids HCl, H_2_SO_4_, carboxylic acid, citric acid and nitric acid have been utilized for the production of highly pure amorphous silica from rice husks and oil palm ash [33,34,35]. TEOS is a Sol-Gel precursor for the production of silica-based nanomaterials, because it is able to bond with polymers via the creation of a link between the hydroxyl group of polymers and silanol groups through covalent and hydrogen bonds [36]. This research motivated us to use alternative sources of production of silica nanoparticles.

Coir pith is a by-product of padding that is employed in mattress factories. It is a lignocellulosic biomass that is produced during the extraction of coir fiber from coconut husk [37]. It has a huge amount of lignin. It accumulates near coir processing industries as a waste product and caused environmental and disposal complications. The raw coir pith comprises 30% lignin, 26.5% cellulose, 26% carbon and 17.5% others [38]. Hence, this investigation focused on the usage of coir pith in the production of nanosilica.

Following the abovementioned, our hypothesis addressed whether it might be environmentally and techno-economically reasonable to produce _n_Si via the acidic Sol-Gel method (biopolymer and TEOS as Si precursor) as a tool to improve seed germination (shoot and root formation, in particular).

## 2. Materials and Methods

### 2.1. Extraction of Lignin Form Coir Pith

Raw coir pith was obtained from the coir-processing industry. It was cut 2 cm, and the biomass obtained was immediately washed under running tap water for about 5 min and then with 20 L of distilled water until no impurities remained. A total of 20 g of powdered coir pith was treated with 200 mL of toluene–ethanol (2:1 ratio (*v*/*v*)) for dewaxing process. Then lignin was extracted from dewaxed coir pith through the alkali extraction method. After that, a dark brownish solution was attained and separated via filtration. Next, the filtrate was concentrated by using a hot-air oven at 60 °C. The ethanol and HCl were mixed to remove the soluble cellulose. The extracted lignin was kept at −4 °C for production of nanomaterials. The amount of lignin (24.5 ± 2.5 g/kg) from the coir pith was assessed by using the protocols of Periakaruppan et al. [39]. 

### 2.2. _n_Si Synthesis

A total of 100% of coir pith mediated lignin (obtained according to Section 2.1) was mixed with 12.5 mL of Tetraethyl orthosilicate (99% purity, CAS 78-10-4, MERCK Inc., Darmstadt, Germany) as a precursor and ethanol (95%, MERCK Inc., Darmstadt, Germany) and continuously stirred for 10 min at room temperature. Then 1 M HCl (99% purity, CAS 30827-99-7, MERCK Inc., Darmstadt, Germany) was added to the mixture and slowly stirred for 15 min at room temperature. At the end, the jelly-like precipitation was formed. The precipitate was kept for 10 h in an EKOCELL drier (MMM Group, Planegg, Germany) at 90 °C for drying. The white-color powder was obtained at the last, and it was stored in an airtight plastic container for further studies [19] (Al-Azawi et al., 2019). Here, lignin and its monomers acted as reducing and capping agents for the formation of silica nanoparticles. Lignin and TEOS were reacted and formed as silica nanoparticles through polymerization. Lignin was an effective chelating agent.

### 2.3. _n_Si Characterization

Lignin-mediated _n_Si was slowly dissolved (1:10) in distilled water of analytical purity and sonicated (five 20 kHz cycles) via UP100H ultrasonic homogenizer (Hielscher Ultrasonics, Teltow, Germany) via the at room temperature. The absorption maxima of _n_Si solution were determined by the C10082MD (Hamamatsu, Japan) UV–Visible spectrophotometer from 200 to 800 nm. Then _n_Si was placed on the quartz slide, and then the IFS 66v/S vacuum FTIR spectrometer (Bruker optics, Woodlands, TX, USA) was employed to observe the FTIR spectra of materials in the range of 4000–400 cm^−1^. An X-ray diffractometer (XRD) was used to analyze the nature of _n_Si. The sizes of the _n_Si samples were calculated by the Scherrer’s formula. The surface morphology of synthesized silica nanoparticles was observed by using the JSM–7610F Schottky field emission scanning electron microscope (JEOL, Tokyo, Japan). Epsilon Xflow (Malvern P Analytical, Ltd., Malvern, United Kingdom) energy-dispersive X–ray analyzer (EDAX) was used to find out the elemental composition (atomic weight percentage of elements) of the _n_Si. The zeta potential of the _n_Si was determined by the measurement of the electrophoretic mobility, using the LS 13 320 XR (Beckman Coulter, Inc., Pasadena, CA, USA) particle-size analyzer. The thermal stability of synthesized silica nanoparticle was assessed by TGA/DSC 3+ (Mettler Toledo, Columbus, OH, USA) thermogravimetric analyzer with a small furnace. 

### 2.4. Agricultural Application

Seeds of *V. unguiculata* were obtained from a local botany garden. The collected seeds were surface sterilized with Savo Original (Unilever, Prague, Czech Republic) liquid disinfectant and washed three times with distilled water. Four different concentrations (25%, 50%, 75% and 100%) of _n_Si were prepared by using distilled water and denoted as T1, T2, T3 and T4, respectfully. The control (distilled water) was maintained and referred to as T5. Then, sterile Petri dishes were taken, and 10 seeds were placed on them. The 5 mL of respective concentration of _n_Si was poured on the corresponding Petri plates. Next, all of the plates were placed at room temperature in dark condition for 3 days. Three independent replications were made for this study. Seeds with a root tip of 1 cm and higher were considered as the germinated seeds. Lengths of roots and shoots (in cm) were observed after 3 days of incubation. After germination, the lengths of roots and shoots of *V. unguiculata* were measured.

## 3. Results and Discussion

### 3.1. Physiochemical Characterization

#### 3.1.1. Analysis of Optical Properties

**Figure 1:**
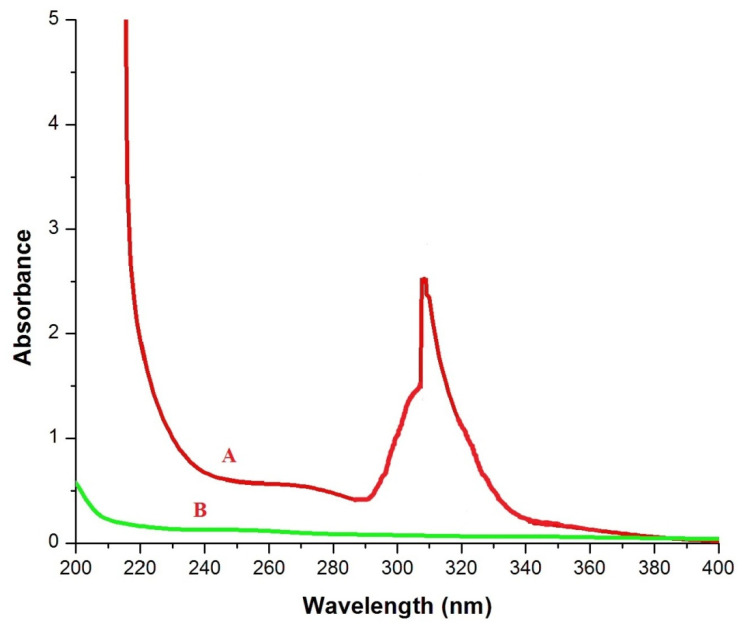
UV spectra, where A = _n_Si and B = extracted lignin from coir pith. (Optical property was assessed).

The UV–visible absorption spectra of _n_Si were recorded as depicted in Figure 1. The absorption spectra of _n_Si were found to be 280–350 nm. The bandgap vibration of electronic transition was found at the broad value of 305 nm for soluble silica suspension. In line with this, Patil et al. [40] concluded that the UV–visible spectrum of _n_Si displayed the maximum absorption band edge of 310 nm. The optical property of silica nanomaterials is related to the occurrence of various defects caused by the partial formation of a Si-O-Si tetrahedral network at the surface, namely silicon and oxygen vacancies.

#### 3.1.2. Analysis of Functional Groups

**Figure 2:**
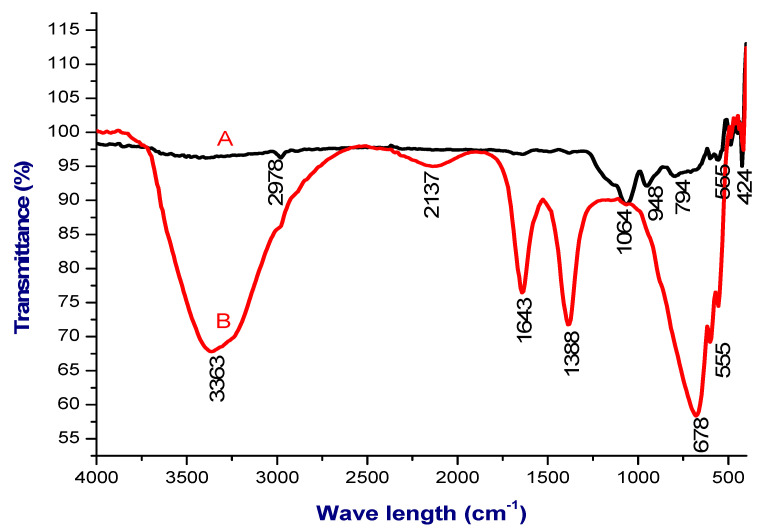
FTIR spectra, where A = _n_Si and B = extracted lignin from coir pith. (Metal oxide group of the _n_Si was observed).

FTIR characterization is routinely used to identify the molecules and their functional group present in the synthesized _n_Si. As shown in Figure 2, the FTIR spectra of lignin displayed different peaks at 3363, 2137, 1643, 1388, 678 and 555 cm^−1^, whereas the FTIR spectrum of the _n_Si produced successive absorption peaks at 1064, 948, 794, 555 and 424 cm^−1^. The oxide group of _n_Si was observed at 794, 555 and 424 cm^−1^, respectively. The FTIR analysis concludes that the formation of silica nanoparticles through the presence of asymmetric stretching vibration of Si-O-Si at 3363 cm^−1^ and another peak at 948 cm^−1^ refers to Si-OH bond. A peak at 424 cm^−1^ corresponds to Si-O-Si bending (Figure 2). Shoulder (Si-O-Si) asymmetric stretch was observed at a peak of 1064 cm^−1^. The bands at 794 and 948 cm^1^ are connected with the complex Si-O-Si symmetric bond stretching vibration. A peak of 2978 cm^−1^ denotes the C=O vibrations. An absorption peak at 1643 cm^−1^ corresponding to the amide I bond of proteins formed due to carbonyl stretch (Figure 2B). Yadav et al. [41] and Imoisili et al. [42] reported similar FTIR signals for their _n_Si.

#### 3.1.3. XRD Analysis

**Figure 3:**
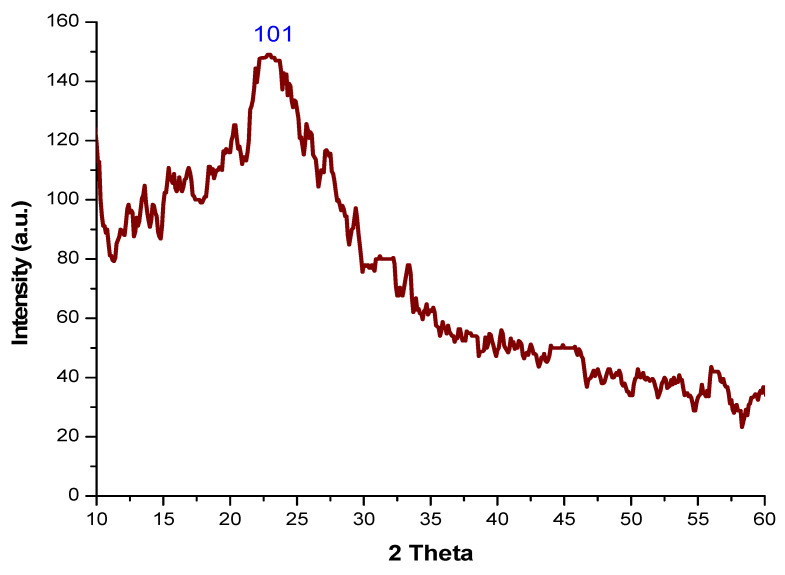
XRD analysis of _n_Si.

XRD was used to determine the structure of nanoparticles. Figure 3 shows the XRD analysis of chemically synthesized _n_Si. The XRD pattern of _n_Si displays a strong narrow and sharp peak, indicating that the _n_Si samples obtained are of high quality and have an amorphous nature. The XRD of the _n_Si revealed the characteristic peaks at 101 planes and the amorphous nature at a diffraction angle of 2ϴ = 20°. The average size was calculated by using the Scherrer equation (D = KλβCosθ^−1^), where *D* is the size, *λ* is the wavelength of X-ray, *θ* is the Braggs angle (in radians) and *β* is the full width at half maximum of the peak (in radians). The average size of synthesized _n_Si is 18 nm. Similarly, Rojas et al. [43] reported that their Si synthesized from rice husk exhibited a most prominent peak at 2θ = 22.01°, corresponding to the (101) plane. The silica nanoparticles produced by Ghani et al. [44] had an amorphous nature and It was confirmed through XRD analysis. Silica nanoparticles were synthesized by using the raw materials of rice hulls in a simple and inexpensive method, and synthesized silica nanomaterials had an amorphous structure [45]. The amorphous structure of nano-SiO_2_ was predicated by using the XRD technique [46].

#### 3.1.4. SEM Analysis

**Figure 4:**
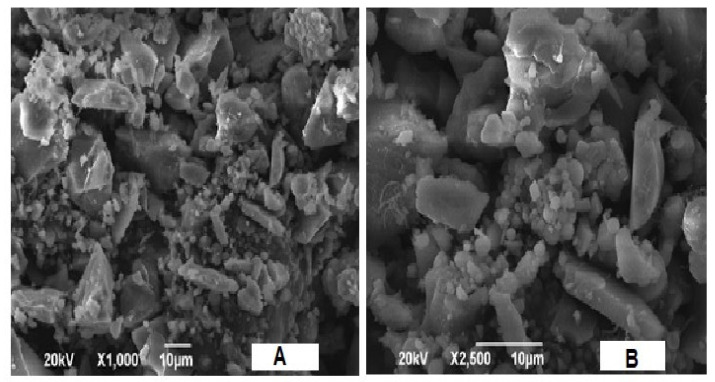
SEM images of _n_Si. (Spherical-shaped _n_Si was observed.) (**A** = X1000 and **B** = X2500).

The SEM image of _n_Si synthesized by using extracted lignin from coir pith is presented in Figure 4. The newly proposed method revealed the monodispersed distribution of particle sizes in the surface morphology, as well as the size of _n_Si. The images display the spherical nature of _n_Si. It depicts mostly spherical _n_Si specimens, as well as the number of aggregates, and some of them represent nanoparticles with an undefined shape. The results presented are corroborated with Verma et al. [47], who synthesized the spherical-shaped _n_Si, and the morphology was indirectly confirmed by using SEM analysis.

#### 3.1.5. EDAX Analysis

**Figure 5:**
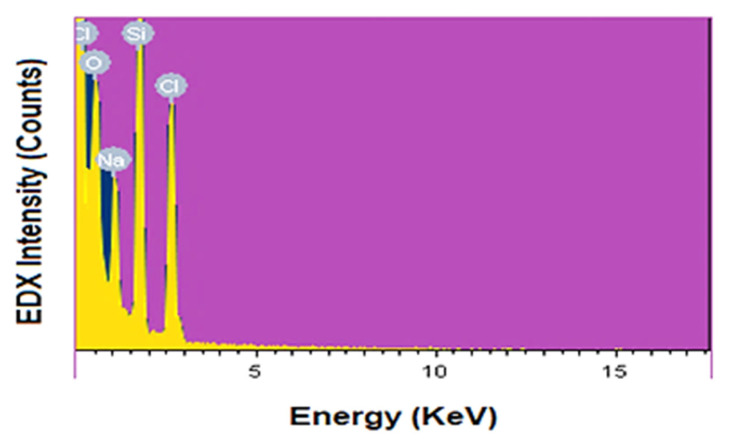
EDX spectra of _n_Si. (Purity and weight of elements were assessed).

EDAX analysis confirms the purity of the _n_Si formed by extracted lignin from coir pith. Figure 5 displays the spectra of _n_Si, using energy-dispersive X-ray spectroscopy (EDAX). Only the signal peaks corresponding to Si (25.58%), O (41.58%), Na (12.12%) and Cl (20.72%) (Table 1) were visible in the spectra. The EDX spectrum obtained shows that the peaks refer to silica and oxygen, indicating that the prepared nanoparticles are silica. From the results, it was confirmed that impurity elements, such as sodium and chloride, are present in the samples. Na and Cl were derived from partially purified lignin because lignin from coir pith was purified and concentrated by HCl and NaOH. In the same manner, Kao et al. [48] observed a closely related result of _n_Si synthesized from chemical mechanical polishing (CMP) steel substrate and reported that the nanoparticles that resulted were clearly composed of Si and O elements. 

#### 3.1.6. Analysis of Zeta Potential and Thermal Stability 

**Figure 6:**
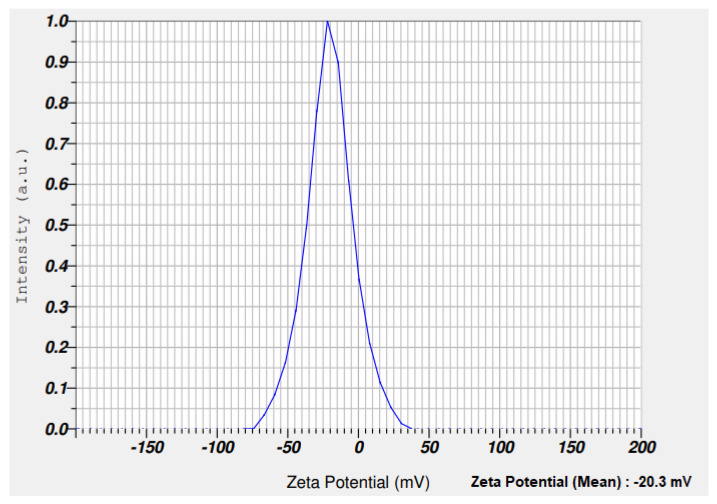
Zeta potential analysis of _n_Si. (Stability of _n_Si was assessed, and _n_Si has a negative charge).

**Figure 7:**
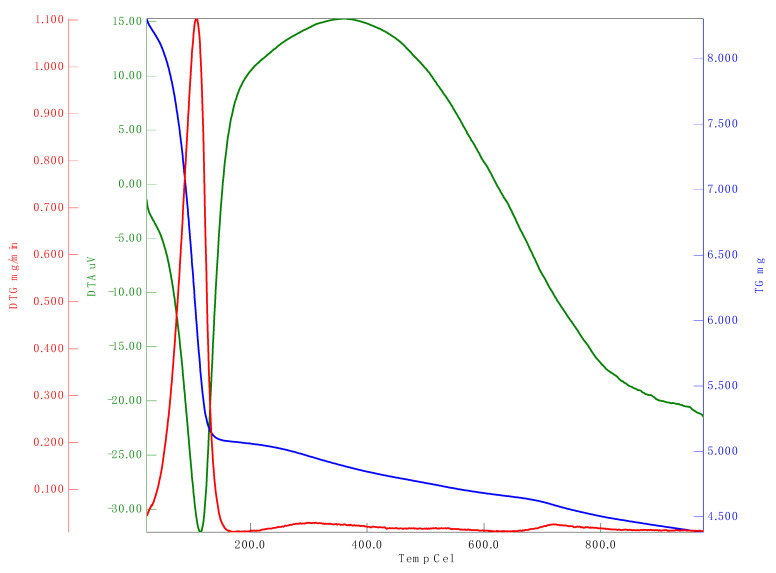
TGA analysis of _n_Si. (Weight loss at different temperatures was observed.) Red line, DTG; blue line, TG; green line, DTA.

Zeta potential analysis is used to assess the electrophoretic mobility of nanomaterials. The zeta potential of synthesized _n_Si was –20.3 mV (Figure 6). The synthesized nanomaterials have a negative charge and are highly stable. Babu et al. [49] demonstrated the green approach for the synthesis of _n_Si from *Cynodondactylon*. They performed the zeta potential studies and reported a zeta potential value of _n_Si(−23.3 mV). Figure 7 refers to the spectrum of thermal stability for the synthesized _n_Si obtained. A 35% weight loss occurred at 150 °C, and gradually the weight reduced up to 45% at 1000 °C. It shows that totally 45% of weight loss appeared between 100 and 1000 °C in _n_Si. The weight loss was attributed to the loss of organic solvents and hydroxyl groups from _n_Si (it was investigated by the exothermic peak in the DTA curve), where the silanol groups were dehydrated at the end. Ethanol was employed during the synthesis of _n_Si. The hydroxyl group were fabricated with _n_Si, and the loss occurred between 50 to 150 °C. The weight of _n_Si was stable at the range of 200–1000 °C. The result clearly determined that the reduction of weight loss occurs at increased heat treatment. The weight loss occurred as a result of the decomposition and evaporation of the organic content of the modified silica nanoparticles [50]. The loss of residual organic solvent and physisorbed water from lignin-mediated silica nanoparticles occurred at 150 °C. These results clearly reveal that the degree increase resulted from the reduction of the amount of the modified nanosilica [50].

#### 3.1.7. Market Analysis

The market analysis shows that wholesale pricing of _n_Si of similar characteristics is somewhere in the range of 2.4 up to 3.3 € g^−1^. The cost breakdown (Table 2) shows that the production cost of the _n_Si obtained by this technology might be expected to be around 1.3 €g^−1^, from which a high degree of cost competitiveness can be expected [51]. 

### 3.2. Agricultural Application

With regard to the industrial application, it is evident that the seed germination and shoot and root length of *V. unguiculata* were improved by the lowest concentration of _n_Si (Table 3). The maximum seed germination was obtained after 72 h. The root and shoot lengths of the seedling were significantly higher in the T1 treatment (25% of _n_Si), whereas the minimum seed germination was observed for the T3 treatment (75% of _n_Si). The shoot and root lengths were minimized at a high concentration of _n_Si-treated *V. unguiculata.* The lowest concentration of silica nanoparticles stimulated the biochemical metabolism for better seed germination and root and shoot formation. The highest concentration of nanoparticles inhibited the seed germination through the blocking of the biochemical metabolism. Similarly, the elongation of the root and shoot, the relative water content (RWC) and the activity of photosynthetic pigments were enhanced in *Zea mays* by the treatment of silica nanoparticles [52].

## 4. Conclusions

A method to synthesize _n_Si without using harmful chemicals was demonstrated. The _n_Si were synthesized by using lignin and TEOS as silica precursors. Lignin acts as a capping, stabilizing and reducing agent for the synthesis of _n_Si. 

The amorphous nature of _n_Si was confirmed by XRD analysis, and no crystalline phase was observed. The fabrication of _n_Si occurred due to crosslinking of lignin. 

FTIR analysis of _n_Si revealed the formation of a Si-O-Si symmetric bond. A 45% of weight loss was observed by TGA analysis, due to the evaporation of the organic content of the modified _n_Si.

Characteristics by UV–visible spectroscopy, XRD, SEM, EDAX, FTIR and zeta potential analysis confirmed that the _n_Si specimens obtained meet all of the established commercial standards (average nanoparticle size obtained is 18 nm).

In addition, the low concentration of _n_Si was found to enhance the germination of seedling. Taking into account that the production costs are also lower than the conventional chemical and physical methods, it can be assumed that the presented technology hides an interesting commercial potential. This synthesis condition is so fascinating from the economical point of view for mass production of _n_Si in industrial scales.

## Figures and Tables

**Table 1:** Elemental composition for _n_Si.

S. No.	Elements	Weight %
1	O K	41.58
2	Na K	12.12
3	Si K	25.58
4	Cl K	20.72
	Total	100

**Table 2:** Cost breakdown of the _n_Si production.

Item	Cost Related to Production of 1 g of _n_Si (€)
Feedstock and processing	0.1
Reactants	0.3
Energy	0.2
Equipment depreciation	0.4
Labor	0.2
Directing and others	0.1
Total	1.3

**Table 3:** Seed-germination analysis.

Treatment	_n_Si Concentration	Seed Germination (%)	Shoot Measurement (cm)	Root Measurement (cm)
T1	25%	80	4.7 ± 0.2	1.5 ± 0.2
T2	50%	65	2.0 ± 0.1	0.7 ± 0.1
T3	75%	40	1.0 ± 0.2	0.5 ± 0.1
T4	100%	30	No shoot formation	No root formation
T5	-	80	4 ± 0.2	1.0 ± 0.1

## Data Availability

Data available on request due to restrictions eg privacy or ethical.
